# Antipsychotics and delirium: When to start and what to select

**DOI:** 10.1192/j.eurpsy.2024.102

**Published:** 2024-08-27

**Authors:** G. Stoppe

**Affiliations:** MentAge, Basel, Switzerland

## Abstract

**Abstract:**

Antipsychotics are among the substances that are very frequently used for elderly people and dementia patients in particular. This is known from studies both in outpatient care and in nursing homes. They are often part of a polypharmacy. This group of substances is discussed in the context of the increased risk of falls, increased mortality and also - as here - in the context of the development of delirium.

On the other hand, antipsychotics are drugs for the treatment of delirium, whereby the question of their significance in modern delirium treatment is being asked anew. In the past, butyrophenones in particular have played a role here, partly because of their variable form of administration and also because of their low cardiac impact.

In the context of delirium prevention, the aim is to reduce the number of drugs on the one hand and the anticholinergic load of the drugs on the other. Algorithms and recommendations exist for this.

In the treatment of delirium, the focus is rightly placed primarily on non-pharmacological management. The use of antipsychotics should be reserved for severe states of agitation or agitation in the context of delirium. In other cases, careful judgement is required.

**Disclosure of Interest:**

None Declared

